# SOURCES OF STIGMA AND THEIR IMPACT ON ALASKA NATIVE ADRD CAREGIVERS' WELLBEING

**DOI:** 10.1093/geroni/igac059.189

**Published:** 2022-12-20

**Authors:** Steffi Kim

**Affiliations:** University of Minnesota, Minneapolis, Minnesota, United States

## Abstract

Challenges such as isolation, scarce resources, and limited knowledge of the disease are often the result of stigmatizing experiences from multiple systemic sources. No studies have investigated the impact of sources of stigma on the quality of life in Alaska Native (AN) ADRD caregivers. This exploratory, mixed-method study within a community-based participatory research framework assessed the experience of family stigma among 40 AN caregivers of people with ADRD across Alaska by administering a measure of systemic stigma and describes the impact of stigmatizing experiences on AN caregivers’ quality of life to develop preliminary data-driven stigma-reducing initiatives. AN caregivers completed the Family Stigma – Alzheimer’s Disease Scale (FS-ADS), assessing caregiver stigma, layperson stigma, and structural stigma. Quality of Life was assessed with the Goodness of Life for Every Alaska Native (GLEAN) scale. Preliminary data on structural stigma and its impact on caregiver quality of life will be presented.

